# Effects of age on the neural correlates of auditory working memory in cochlear implant users

**DOI:** 10.1371/journal.pone.0325930

**Published:** 2025-06-25

**Authors:** Priyanka Prince, Claude Alain, Joseph Chen, Trung Le, Vincent Lin, Andrew Dimitrijevic

**Affiliations:** 1 Evaluative Clinical Sciences Platform, Sunnybrook Research Institute, Toronto, Ontario, Canada; 2 Department of Physiology, University of Toronto, Toronto, Ontario, Canada; 3 Rotman Research Institute at Baycrest Academy for Research and Education, Toronto, Ontario, Canada; 4 Department of Psychology, University of Toronto, Ontario, Canada; 5 Institute of Medical Sciences, University of Toronto, Ontario, Canada; 6 Music and Health Science Research Collaboratory, University of Toronto, Ontario, Canada; 7 Otolaryngology-Head and Neck Surgery, Sunnybrook Health Sciences Centre, Toronto, Ontario, Canada; 8 Faculty of Medicine, Otolaryngology-Head and Neck Surgery, University of Toronto, Ontario, Canada; Johns Hopkins University, UNITED STATES OF AMERICA

## Abstract

Following a conversation in a noisy environment is challenging, especially for individuals who received cochlear implants (CIs) to remediate severe-to-profound hearing loss. CI users likely work harder than their normal-hearing counterparts to understand speech in adverse listening conditions. Recruiting cognitive resources can be taxing and may interfere with attentional regulation and working memory processes. Few studies have, however, examined the impact of CIs on the neural correlates of attention and working memory. We used a high-density electroencephalogram to investigate behavioural and neural correlates of auditory attention and working memory in 14 CI users and age-matched normal hearing (NH) controls (age ranges: 21–75). All participants completed an auditory n-back task with zero- and two-back memory load conditions. Behaviourally, CI users were slower in identifying targets during the two-back condition, especially older adults. The sensory-evoked responses were reduced in CI users compared to NH. With increasing memory load, younger CI users and NH controls displayed decreased amplitudes, while older CI users showed no change in amplitude. We also observed reduced frontal theta synchrony and greater alpha/beta desynchrony in CI users where alpha/beta in the left inferior frontal gyrus was related to slower response times in CI users. Attenuated frontotemporal connectivity was also evident compared to NH. These findings suggest that CI users adapt to higher task demands by allocating more attentional resources while encoding and maintaining stimuli in working memory. This pattern of activity potentially indexes a delay in identification and possible neural correlates of cognitive deficits while aging with a CI.

## Introduction

Hearing loss is considered the most significant modifiable risk factor for dementia in older adults, accounting for 8% of global dementia cases [1]. The relationship between hearing loss and decline in various cognitive domains (e.g., attention, memory, executive functions, and perceptual-motor control), however, remains an open question. According to the information degradation hypothesis, hearing loss results in diminished auditory processing that must be thwarted by engaging additional attentional resources and compensatory strategies required for successful communication [2–6]. This leads to increased cognitive effort for a prolonged period and can lead to detriments in quality of life through social isolation, depression, and, over time, cognitive decline [7–17]. In these cases, the remediation of hearing loss should improve cognitive abilities and reduce the incidence of mild cognitive impairment and dementia.

Longitudinal studies investigating hearing loss interventions for dementia prevention allude to this by showing improvements in cognitive functions following hearing restoration with hearing aids (HA) [18–21] and cochlear implants (CI) [22–26]. In many instances, improvements are more prevalent when HA and CI users were cognitively impaired before the intervention. One longitudinal study by Mosnier and colleagues [27] showed preserved cognitive functioning after obtaining a CI. However, mild cognitive impairment was still evident in some post-operative participants who presented it preoperatively (19 out of 31), with a few developing dementia (2 out of 31). Furthermore, some participants with preoperative normal cognitive functioning developed mild cognitive impairment after obtaining a CI (12 out of 38). When compared to normal hearing (NH) controls, CI users, with at least one year of CI experience, exhibited lower performance on cognitive-related tests [28,29] and, with at least six months of CI experience, reported lower quality of life [30]. Therefore, despite the hearing improvement a CI provides, cognitive abilities and quality of life metrics do not appear to improve to the level of NH individuals.

It is difficult to ascertain the reasons behind differences in cognitive performance across CI users purely through behavioural methods. Findings from electroencephalography (EEG) studies have shown that CI users may utilize compensatory mechanisms while encoding verbal stimuli by allocating cognitive resources to perform speech-related tasks [31–34]. These compensatory mechanisms are pivotal to counteract the diminished auditory input in CI users.

Distinct patterns of neural activity have been associated with increased use of cognitive effort and cognitive impairment. For instance, reduced theta synchrony has been associated with fatigue over time [35], lower cognitive performance [35–44], progressive mild cognitive impairment [45–48] and older age [49], implying a deficit in memory and executive functioning. Increased cognitive effort has been related to alpha and beta desynchrony, suggesting an increased use of attentional resources [34,38,50–56]. Similarly, greater alpha activity in the left inferior frontal gyrus (IFG) has been associated with cognitive effort in response to listening to degraded verbal stimuli and speech in background noise [57,58]. In line with these findings, greater alpha/beta desynchrony was observed in CI users, compared to NH, when encoding visual (i.e., numbers and letters) stimuli [34]. Moreover, left IFG activity correlated with subjective listening effort in CI users during a speech-in-noise task [31]. However, to our knowledge, comparing neural correlates of auditory working memory between CI users and NH controls has not been investigated.

This study compares neural activity in CI users and NH controls during an auditory n-back task, which involves updating and maintaining ongoing auditory stimuli while inhibiting irrelevant stimuli [59]. Using EEG, we can observe patterns of activity associated with increasing memory load in CI users. We test the following hypotheses: (i) CI users will exhibit lower performance and slower reaction times during both n-back conditions compared to NH controls; (ii) CI users will exhibit a reduced theta and greater alpha/beta activity compared to NH controls; (iii) CI users will exhibit a larger left IFG activity compared to NH controls; and (iv) relationships between neural activity, demographics, and behavioural performance will be evident in CI users wherein reduced theta is related to older age and lower behavioural performance and greater alpha and left IFG will be related to lower SIN and behavioural performance.

## Methods

### Participants

Fourteen CI users, with no known underlying neurological conditions, were recruited from the patient population in the Department of Otolaryngology at Sunnybrook Health Sciences Centre (age range 21–75, M = 56.1, SD = 18.2; six males and eight females) from April 12^th^, 2022 to January 14^th^, 2023. Demographic information is shown in Table 1. The CI group consisted of four bilateral and ten unilateral users. Among the unilateral users, eight used a hearing aid on their contralateral ear. Speech perception in noise (SIN) scores were measured using the AzBio test [60] as part of their standard clinical testing. The scores we obtained were administered one year or more after the activation of their CI at an SNR of +5 dB; these scores were used for correlational analyses in addition to age, age of implantation, duration of deafness (time before obtaining a CI) and duration of implantation. Fourteen age-matched controls were also recruited with ages ranging from 21–75 years (M = 53.9, SD = 17.2) and included ten males and four females with no known underlying neurological conditions. They were recruited through local databases and online social media groups in Toronto, Canada.

**Table 1:** Demographic of CI Users.

CI Participants	Sex	Age	Condition	AzBio in Noise (+5)	Etiology
1	M	21	Bilateral	83	Congenital
2	M	24	Bilateral	10	Congenital
3	F	31	Unilateral (Right CI Left HA)	8	Genetics/Progressive since childhood
4	F	51	Unilateral (Right CI Left HA)	100	Genetics/Progressive as adult
5	F	54	Unilateral (Right CI)	56	Usher’s Syndrome
6	F	55	Unilateral (Right CI Left HA)	64	Illness/Progressive since childhood
7	M	64	Unilateral (Right CI Left HA)	65	Unknown/Progressive as adult
8	F	65	Unilateral (Left CI Right HA)	79	Congenital/Progressive since childhood
9	M	66	Unilateral (Right CI Left HA)	17	Genetics/Progressive as adult
10	M	69	Bilateral	18	Cogan’s Syndrome
11	M	69	Unilateral (Right CI Left HA)	29	Unknown/Progressive since childhood
12	F	70	Unilateral (Right CI Left HA)	41	Meniere’s Disease
13	F	72	Unilateral (Left CI)	35	Unknown/Progressive as adult
14	F	75	Bilateral	0	Meniere’s Disease

All participants provided written and informed consent in accordance with the Research Ethics Board at Sunnybrook Health Sciences Centre. The approved protocol was in accordance with the Declaration of Helsinki. Participants were monetarily compensated for their participation and were reimbursed for parking fees at the hospital campus.

The individuals are numbered 1–14, from youngest to oldest. Their corresponding sex and age are recorded, their condition (bilateral, unilateral, and/or if a hearing aid, HA, is used, specifying left or right ear lateralization), outcomes of their speech perception tests and etiology of their condition, respectively from left to right.

### N-Back working memory task

**Fig 1:**
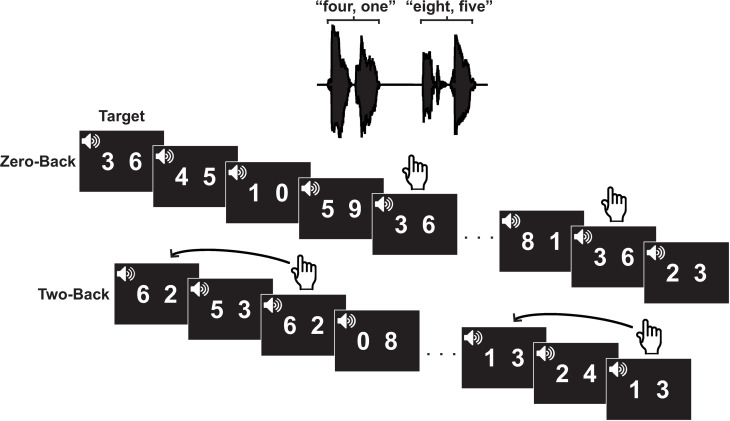
Example of each condition. Auditory representations of the double-digit numbers are displayed on top. Zero-back is shown in the middle where the target is presented first then the sequence of ten double-digit numbers. Two-back is displayed at the bottom in which a sequence of ten double-digit numbers is presented containing the targets defined as numbers presented two positions earlier. Black squares indicate the numbers played auditorily through a speaker in front of participants (0^o^ angle). Pointer fingers indicate when the target is presented and when button press should occur.

Participants performed two conditions of the n-back task: zero- and two-back (Fig 1). For each condition, the stimuli were double-digit numbers presented with the numbers spoken individually and the numbers were played from a pre-recorded database. The numbers were from zero to nine and randomly paired for each trial; seven was excluded since it contains two syllables. The zero-back condition was a “low” memory load. In this condition, participants were presented with “0-Back: Listen” on screen while a target number was played auditorily. Their task was to press a button in response to the occurrence of the target within a sequence of ten double-digit numbers. The two-back task was the experimental “higher” working memory condition where the instruction “2-Back” was presented visually. Then, participants were presented with a sequence of ten double-digit numbers comprising of targets defined as number pairs played two positions earlier in the sequence. Participants were told to focus their gaze on a visual crosshair on the center of a computer screen while the numbers were presented through a speaker located at 0-degree azimuth in front of them at a level of 65 dB SPL. The targets were presented 1–4 times in each trial and participants were tasked to respond to the targets accurately and as quickly as possible. Performance was calculated based on target identification, and response times (RTs) were calculated by the time between the onset of the second digit and the button press. Each condition consisted of 100 trials separated into four blocks of 25; therefore, participants completed 200 trials.

### EEG Recording and Preprocessing

The EEG was recorded and preprocessed using Brain Vision Analyzer software [61], sampled at 2 kHz using a BrainAmp DC amplifier from 64 equidistant sensors on an ActiCAP, and referenced online to the vertex electrode. The equidistant layout covers a larger area than a standard 10–20 system to improve source localization estimates [31,56]. Each participant’s 3D surface electrode positions were digitally mapped using a Polhemus Patriot (Colchester, VT, USA). Approximately 1–3 sensors around the temporal regions near the magnet and coil of the CI were not recorded. Spline interpolation replaced these channels with derived estimates based on activity from neighboring sensors and included in the analyses.

Raw EEG data were filtered from 0.1 to 40 Hz through a 2nd-order Butterworth filter, then downsampled to 250 Hz. Continuous data were then subjected to independent component analysis (ICA) to identify myogenic artifacts (e.g., eye blinks and eye movements) and other contaminants (e.g., intermittent faulty electrodes). Visual inspection confirmed artifactual noise, and the corresponding independent component weights were set to zero before the EEG was reconstructed. We were cautious in removing only components containing the artifact; between five and eight were removed per participant. Noisy channels were replaced by derived estimates from neighboring sensors using spline interpolation. An additional ICA was performed to identify CI artifacts using components with a centroid of activation on the side of their CI [62,63], where one to three artifacts were removed per participant. After cleaning, continuous EEG data was exported into EEGLAB [64] and Brainstorm for analyses.

### Neural activity of stimulus encoding

#### Sensor level analysis.

Auditory evoked potentials (AEPs) were examined by averaging activity across the target double-digit pairs within each condition. The continuous EEG data was epoched into 2.8-second segments time-locked on the onset of the first digit: 0.5 seconds before the onset and 2.3 seconds after. EEG data for each AEP were re-referenced to the scalp average, and the baseline offset was corrected using the −0.5 to 0-second interval. Trials containing noisy artifacts, not corrected for by ICA, were removed by visually inspecting and by removing trials with any channel exceeding 120 µV. The resulting individual data files were exported from EEGLAB to Fieldtrip [65] in MATLAB (2021a, The Mathworks, Inc., Natick, MA, United States). EEG sensors for analysis of AEPs were chosen based on an inspection of grand average responses across participant groups, and six channels (e2, e3, e4, e33, e34, e35) across the frontocentral region were chosen for analysis and visualization of AEPs. Three AEP components were analyzed for each digit: the first positive going P1 response occurring around 50 ms, an N1 response occurring around 100 ms, and a P2 occurring around 200 ms. For each participant, voltages were averaged across a 20-ms window based on the peaks of their groups’ grand mean responses.

To obtain an average time-frequency representation (TFR) of the event-related EEG, we segmented continuous EEG data into 2.8-second epochs, including 0.5 seconds before the onset of a character (indicated by a visual trigger) and 2.3 seconds after stimulus onset. Evoked responses were averaged across trials and subtracted from each trial in MATLAB before importing data into Brainstorm [66]. This method was used to minimize the oscillatory responses to evoked stimuli to identify induced responses [67]. We used the Fieldtrip plugin in Brainstorm, multitaper time-frequency decomposition [68,69] to compute TFRs with a frequency resolution of 1 Hz from 1 to 30 Hz and a temporal resolution of 250ms.

#### Source analysis of AEPs.

**Fig 2:**
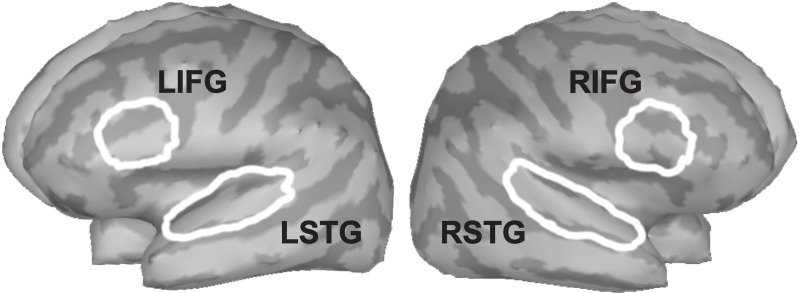
Location of regions of interest (ROI). ROIs are highlighted in white in their respective locations labelled by their abbreviations. The ROIs used were left inferior frontal gyrus (LIFG), right inferior frontal gyrus (RIFG), left superior frontal gyrus (LSTG), and right superior frontal gyrus (RSTG).

Sources of AEPs were obtained, first, by creating a boundary element head model using the OpenMEEG [70,71] plugin in Brainstorm, then standardized low-resolution electromagnetic tomography (sLORETA) modeling [72] was computed using the default settings in Brainstorm [73]. Source analysis of TFR was obtained using the linearly constrained minimum variance vector beamformer, which is suitable for TFR using the Pseudo Neural Activity Index (PNAI). Each sLORETA map was used to extract the absolute values of the source time series (aka “scouts”) in anatomically defined regions of interest (ROIs) based on the Desikan-Killany atlas [74]. The bilateral auditory cortices (superior temporal gyri; STG) were extracted from the sLORETA map and for the TFR, bilateral IFG (left and right) scouts (pars triangularis, opercularis and orbitalis) were extracted alongside the left and right STG from PNAI maps (Fig 2). The IFG was chosen due to its association with cognitive effort during speech-related tasks [57]. Specific windows (time or time and frequency) were extracted from each participant and determined by the significant differences found at the sensor level.

Weighted phase lag index (wPLI) was used to investigate functional connectivity between ROIs [75]. WPLI overcomes problems related to volume conduction in coherence analysis and increases statistical power relative to other phase synchronization options [75,76]. After functional connectivity analyses were performed, brain activation measures were obtained from ROIs (Fig 2). We specifically focused on the connectivity between STG and IFG because previous studies have shown its involvement in encoding verbal stimuli [34,77–82]. Connectivity measures were obtained for the theta and alpha/beta time-frequency windows.

### Statistical analysis

Statistical tests were performed using Brainstorm or R [83]. The accuracy and RTs were analyzed using mixed model ANOVA with groups as the between-subject factor and conditions as the within-subject factor. Post hoc comparisons were completed using the *emmeans* package and were corrected for by false discovery rate (FDR) [84]. The relationship between behavioural outcome measures, demographic variables and clinical SIN scores were examined using correlational analyses from the *psych* package [85].

Sensor level and source activations of AEPs were subtracted between conditions for each digit (i.e., first digit P1 in zero-back minus first digit P1 in two-back). The subtraction of the AEPs elicited by the digits between conditions was performed to control for any possible reduction in neural activity due to ICA in CI users. Sensor-level differences for each AEP component (first and second-digit P1/N1/P2) were compared between groups using unpaired sample t-tests through the built-in functions in R and then corrected for multiple comparisons using FDR. For source-level differences, group and hemispheres (left and right auditory cortex) were compared for each AEP component using mixed ANOVAs. Differences in time-frequency and connectivity data between groups and conditions were analyzed using cluster-based permutation independent and paired t-tests in Brainstorm; this was performed with Monte-Carlo approximation (5000 permutations). Significant neural differences between groups were correlated with behavioural outcome measures, demographic variables and SIN using R. Spearman correlation was chosen because it is less sensitive to outliers compared to Pearson [86]. To test the robustness of the correlations, outliers (when apparent) were removed resulting in non-significant results. However, considering the small sample size of the CI group, it is unclear whether the non-significant results were a consequence of removing the outlier or decreasing the sample size.

All t-tests and Spearman correlations were two-tailed, and the alpha criterion for Type I error was set at 0.05 with effect sizes expressed by partial eta squared (η^2^_*p*_) for ANOVA results and Cohen’s *d* for t-tests.

## Results

### Behavioural results

**Fig 3:**
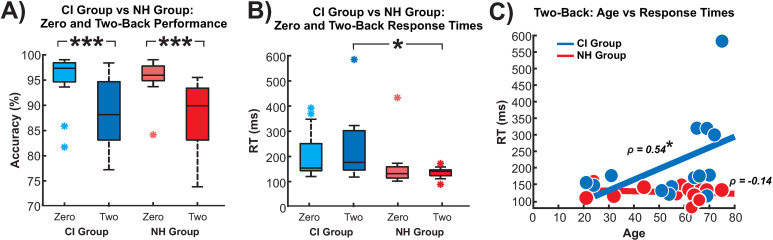
Behavioral measures for CI users and NH controls in during the zero-back and two-back conditions. Condition and group differences in A) performance and B) reaction time. **C)** Correlation between RT during two-back and age for both groups. *** = p < 0.001, * = p < 0.05.

Fig 3 shows CI and NH accuracy and RTs (Fig 3A-B) on zero- and two-back conditions. As anticipated, the zero-back was completed with little difficulty with participants performing near the ceiling (CI: M = 96%, SD = 0.054; NH: M = 96%, SD = 0.038). However, both CI users and NH showed reduced accuracy for the two-back condition (CI: M = 89%, SD = 0.07; NH: M = 86%, SD = 0.068). A 2x2 ANOVA (CI/NH x zero-back/two-back) showed that CI users and NH controls were more accurate in the zero- than the two-back condition and results showed a large effect size (main effect of condition: F(1,26) = 35.05, p < 0.0001, η^2^_*p*_ = 0.57). The main effect of group on accuracy was not significant (F(1,26) = 0.005, p = 0.94, η^2^_*p*_ = 0.0002) nor was the group x condition interaction (F(1,26) = 0.045, p = 0.83, η^2^_*p*_ = 0.002).

The observed RTs to the onset of the second number were shorter than those previously reported in auditory n-back tasks [49,87,88]. Since the stimuli are two-digit numbers, with each number spoken individually, participants would be primed after the first number to decide whether it is the target. For the RTs, a significant main effect for the group was observed with a large effect size (F(1,26) = 4.963, p = 0.035, η2_*p*_ = 0.16), where NH controls were faster than CI users (Fig 3B). The main effect of condition was not significant (F(1,26) = 0.002, p = 0.96, η2_*p*_ < 0.0001) nor was the group x condition interaction (F(1,26) = 1.183, p = 0.29, η2_*p*_ = 0.04).

In CI users, RTs during the two-back were significantly correlated with participants’ age (ρ = 0.54; p = 0.048, FDR corrected) and in NH controls, this relationship between RT and age was not significant (ρ = −0.14; p = 0.64, FDR corrected). The correlational coefficients differed significantly between groups (Fisher’s r-to-z transformation = 1.75, p = 0.04). There were no significant correlations between accuracy and demographic variables.

### AEP sensor and source level activity

**Fig 4:**
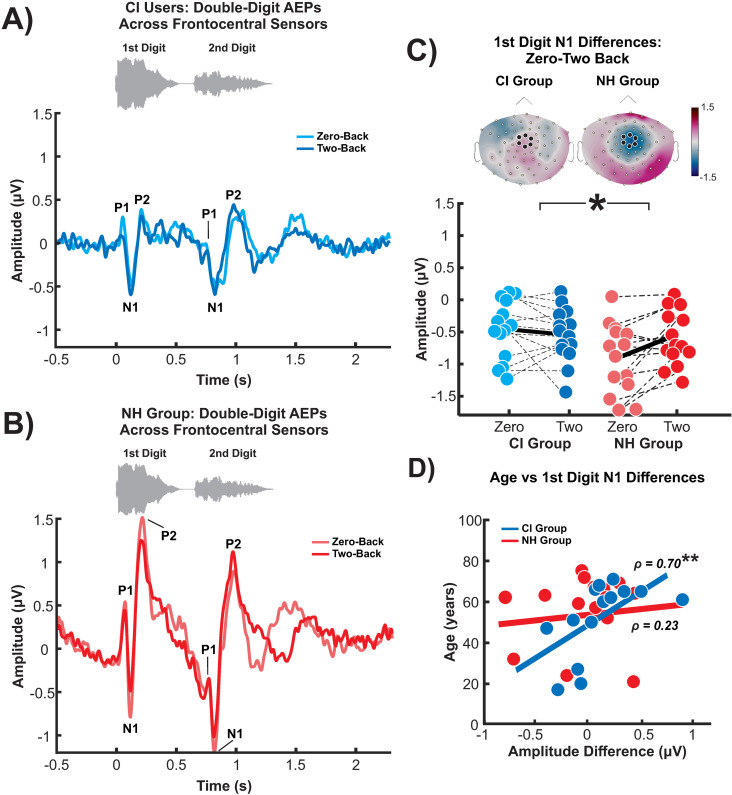
Group mean auditory evoked potentials (AEPs) during the zero- and two-back condition over frontocentral sensors for **A)** CI users and **B)** NH group. **C)** N1 difference between two-back to zero-back compared between groups. Isocontroup maps of the difference in waveforms (0-back minus 2-back) are presented with the frontocentral sensors highlighted; the left is CI users, and the right is NH. CI users and NH controls significant correlations between age and D) first digit N1 amplitude differences subtracted between two conditions. ** = p < 0.01, * = p < 0.05.

Fig 4 shows the AEPs averaged for each group and condition. Two distinct P1-NI-P2 complexes were observed in both groups, one for each digit presented. Reduced AEP amplitudes were observed in the CI users for both conditions compared to NH controls (zero-back: N1 and P2 in both digits; two-back: P2 first digit and P1, N1, and P2 during the second digit; p < 0.05).

Unpaired t-tests were conducted to compare AEP amplitude differences (zero- minus two-back) between groups to determine how memory load affects the encoding of the digit pair. NH controls’ first digit ΔN1 (N1 difference from the zero- to the two-back), compared to CI users, was significantly more negative with a large effect size (t(26) = −2.73, p = 0.01, *d* = 1.03, FDR corrected). Simply put, the N1 amplitude of the first digit was greater in the zero-back for NH controls and was greater in the two-back for CI users. No significant differences were observed with the other AEP components (p‘s > 0.05).

Correlation analyses revealed a significant relationship between ΔN1 and age in CI users (ρ = −0.70, p = 0.006, FDR corrected) but not NH controls (ρ = 0.03, p = 0.93, FDR corrected). These correlations significantly differed between groups (Fisher’s r-to-z transformation = −2.104, p = 0.02), showing that increasing age in CI users is associated with a greater increase in N1 from zero- to two-back conditions compared to NH controls.

AEP amplitude differences between hemispheres were also compared at the source level between groups. There were no significant main effects or group x hemisphere interactions. Lastly, there were no significant correlations between demographics and behavioural results.

### Differences in oscillatory activity

**Fig 5:**
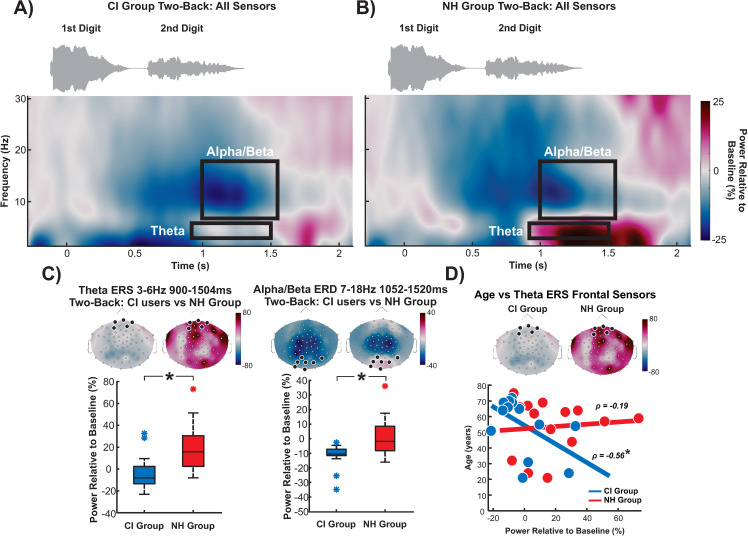
Differences while comparing time-frequency representations during the two-back condition. Significant differences are outlined with black rectangles comparing **A)** CI users and **B)** NH controls. **C)** Theta (left panel) and alpha/beta (right panel) significant differences during the two-back between groups are displayed with their corresponding topographical maps and significant sensors highlighted. **D)** Plot of correlation between frontal theta ERS and age in CI users and NH controls. * = p < 0.05.

After subtracting the evoked responses, time-frequency representations were compared between groups using cluster-based permutation t-tests. The analysis revealed theta and alpha/beta differences between CI users and NH controls during the two-back condition (Fig 5A-B); there were no group differences during the zero-back. CI users showed a significantly diminished frontal theta ERS response compared to NH controls (Fig 5C; p = 0.026). Also, NH controls showed greater alpha/beta ERS over posterior electrodes than CI users (Fig 5C; p = 0.049). Descriptively, while all CI participants displayed an alpha/beta ERD, NH controls showed both ERS and ERD. Theta ERS power was negatively correlated with age such that with increasing age, less theta power was observed in CI users (Fig 5D; ρ = −0.56, p = 0.04, FDR corrected) but not in NH controls (ρ = −0.19, p = 0.51, FDR corrected). However, the theta ERS and age correlations were not significant between groups (Fisher’s r-to-z transformation = −1.03, p = 0.2).

Furthermore, theta and alpha/beta activity were compared between correct and incorrect trials revealing no significant differences (S1 Fig). Additionally, when comparing correct and incorrect trials between groups and conditions, results were also not significant (p’s > 0.2).

**Fig 6:**
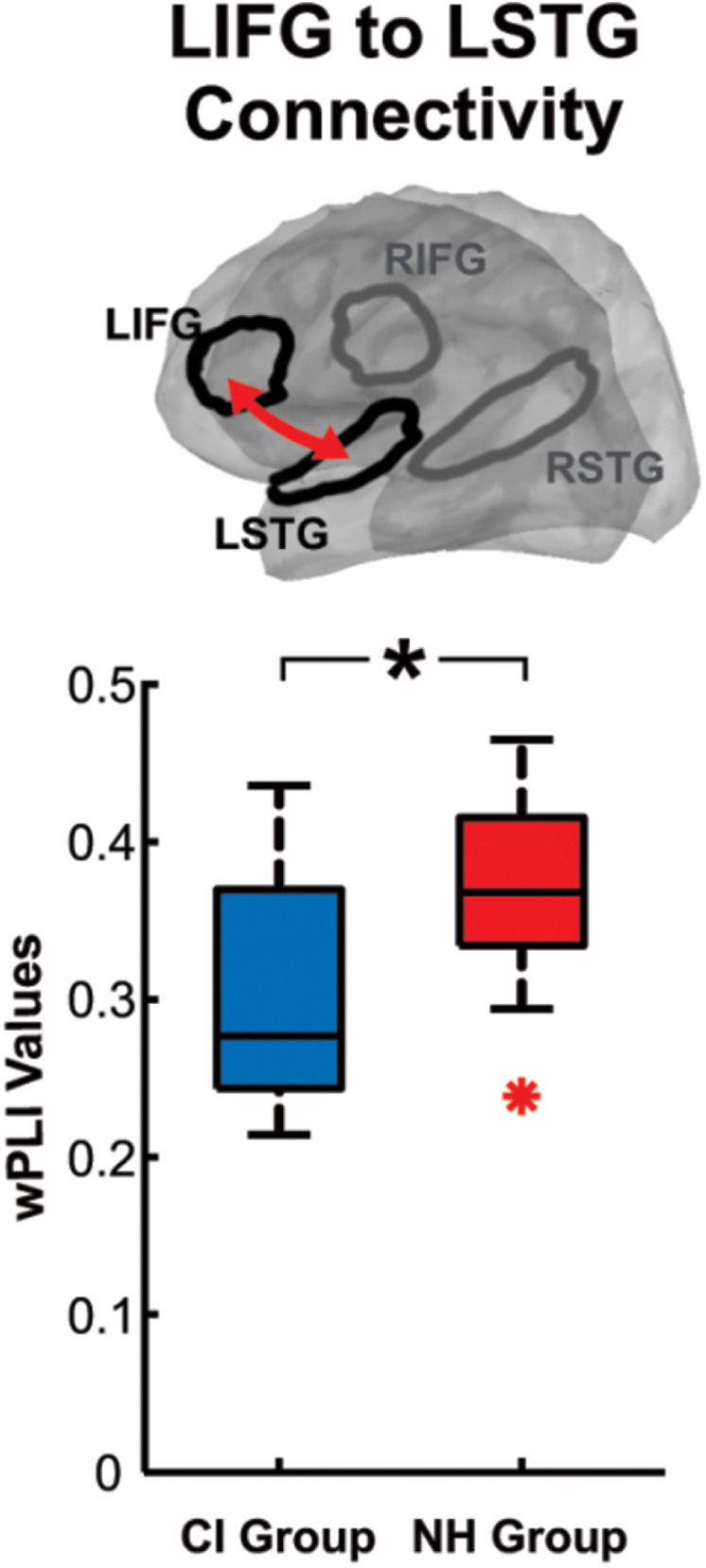
Group mean weighted phase lag index (wPLI) values for CI and NH groups. LIFG = Left Inferior Frontal Gyrus; LSTG = Left Superior Temporal Gyrus. The significant connection between LIFG and LSTG is indicated by the red arrow. * = p < 0.05.

Source activations and connectivity were assessed across the right and left IFG and STG ROIs. As significant differences were observed at the sensor level within the theta and alpha/beta bands, the same time and frequency windows depicted in Fig. 5A-B were used to compare source activations and connectivity between the two groups. The wPLI values indicate the strength of the connection between the ROIs (higher values indicate stronger connections). Although there were no significant source activity differences between groups and no significant group differences in wPLI during the theta window, we observed a significant group difference in wPLI during the alpha/beta window in the two-back condition. CI users showed reduced connectivity between the left IFG and left STG compared to NH controls (Fig 6; p = 0.012). Connectivity between occipital cortices and left IFG were also investigated as an exploratory measure, resulting in no significant differences or correlations. No correlations were observed for the connectivity result; however, significant correlations were observed with the source ROIs.

The source ROI activity and the frontotemporal connectivity metrics were correlated with demographics and behavioural results. CI users with a larger alpha/beta ERD in the left IFG had slower RTs (ρ = −0.65; p = 0.015, FDR corrected) however this correlation was not observed in NH controls (ρ = 0.26; p = 0.37, FDR corrected) and was significantly different between groups (Fisher’s r-to-z transformation = −2.44, p = 0.007). Additionally, older CI users trended towards a larger alpha/beta ERD in the left IFG; however, this finding was not significant in CI users (ρ = −0.49; p = 0.075, FDR corrected) and NH controls (ρ = −0.14; p = 0.63, FDR corrected).

**Fig 7:**
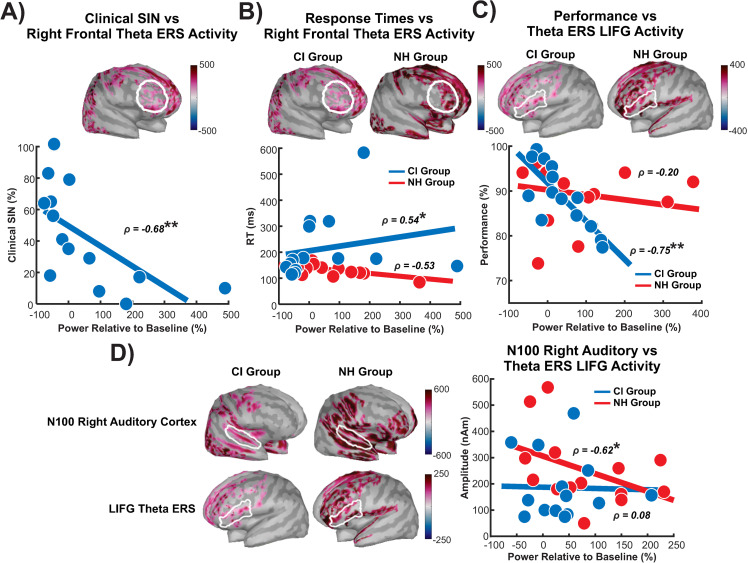
Frontal theta ERS correlations with SIN scores and RTs. **A)** Right frontal ROI encompassing the middle and inferior frontal gyrus is outlined in white and below is a scatter plot of theta ERS activity within this ROI and AzBio scores and **B)** RTs for CI users and NH control. **C)** LIFG is outlined in white and the scatter plot displays the correlation between this ROI and performance for CI users and NH controls. **D)** Right auditory cortex and LIFG is outlined in white and the scatter plot displays the correlation between these two ROIs for CI users and NH controls. * = p < 0.05, ** = p < 0.01.

We conducted exploratory analyses on frontal theta to further understand the observed reduced ERS activity in CI users compared to NH controls. ROIs were created based on frontal activations observed in the grand mean during the theta ERS period which yielded bilateral maxima in the left and right middle frontal cortex. These ROIs correlated with behavioural outcome measures (such as SIN, n-back performance and RTs) and demographic variables. Results were corrected for multiple comparisons through FDR. A correlation between theta activity in the right frontal cortex and SIN in CI users was significant (ρ = −0.68; p = 0.009) after FDR correction (Fig 7A). This time and frequency window (900−1504ms; 3−6 Hz) corresponded with the significant difference between groups observed in the sensor-level data. During the same time-frequency window, greater theta ERS in the right frontal ROI was significantly related to slower RTs (ρ = 0.54; p = 0.048). However, it did not survive FDR corrections. Additionally, greater left IFG theta ERS activation correlated with poorer performance on the two-back condition (ρ = −0.75; p = 0.003), which was significant after FDR corrections. These correlations were not significant in NH controls (p’s > 0.05) and differed significantly from those observed in CI users (p’s < 0.05).

Lastly, frontal theta ERS was correlated with the strength of the N1 sources. The NH controls displayed a significant correlation, where greater right auditory cortex activity during the N1 was related to lower theta activity in the left IFG (Fig 7D; ρ = −0.62; p = 0.02, FDR corrected). This correlation was not significant in CI users (ρ = 0.08; p = 0.80, FDR corrected) and was significantly different compared to NH controls (Fisher’s r-to-z transformation = −1.89, p = 0.03).

## Discussion

### Summary

The main findings of this study were observed in the two-back condition and are as follows: longer RTs were observed in CI users compared to NH controls, however, contrary to our hypothesis, accuracy was similar between the groups. During stimulus encoding, NH controls displayed a decrease in N1 amplitude from the zero- to two-back conditions, while minimal changes were observed in CI users who, as a group, had reduced amplitudes compared to NH. Investigation of oscillatory activity elicited by the second digit revealed reduced frontal theta ERS and greater posterior alpha/beta ERD in CI users. During the alpha/beta time-frequency window, the left IFG was not significantly different between the groups as predicted. However, reduced connectivity between the left IFG and the left STG was observed for CI users compared to NH controls.

Significant correlations across EEG/demographics/behavior metrics were observed: (1) increasing age in CI users related to longer RTs, greater increase in N1 amplitude from zero- to two-back, and diminished theta power. (2) Increasing clinical SIN scores related to decreases in right frontal theta during the two-back condition. (3) Greater accuracy scores were related to diminished left frontal theta power in CI users. (4) Longer RTs in CI users were related to increased right frontal theta power and greater alpha/beta ERD in the left IFG. (5) Lastly, the greater the N1 auditory cortex activation in NH controls, the smaller the theta power in the left IFG. Overall, these correlation results suggest that aging may affect CI users differently than NH controls and that neural differences during stimulus encoding may result in slower RTs, lower accuracy on the n-back task and lower SIN outcomes.

### Working memory performance and RTs

Literature on auditory working memory in adult CI users is sparse and inconsistent. While Tao et al. (2014) and Hamdy et al. (2023) observed lower performance on a digit span task for CI users [89–91], Moberly et al. (2017) reported no differences during the same task [92]; however, this could be related to repeating the sequence length twice to participants. In the present study, we did not observe significant differences in accuracy between groups in the zero- and two-back conditions. However, a delayed RT was observed in CI users compared to NH controls. Similar findings regarding RTs were observed during an auditory lexical decision task [93] and visual word recognition task [94] where CI users yielded slower RTs compared to NH. These suggest a similar ability to identify auditory targets between groups. However, CI users experience a delay in cognitive processing compared to NH controls.

The delays in identifying auditory stimuli are perhaps due to an increased reliance on cognitive factors such as working memory and attention to disambiguate spectrally degraded stimuli caused by listening through a CI [95–98]. Notably, older CI users exhibited more delayed responses to targets, which was not observed in NH age-matched controls. RTs did not relate to the duration of deafness and duration of implantation in CI users, confirming that it is more likely an age effect. Although other n-back studies on NH participants have observed this correlation [99,100], the modality of stimuli presented (visual or auditory) is suggested to influence the age effect where age-related differences are more apparent when stimuli are visually presented [101]. To our knowledge, only one study reported similar findings in CI users in which older adults exhibited slower lexical access [102] while other studies have reported poorer performances in auditory processing [102–104] and cognitive-related factors [102,103,105]. Therefore, older CI users possibly recruit more cognitive resources for speech perception than younger, resulting in delays in processing speech.

### Adaptation to task demands

We compared the adaptation of auditory processing from a manageable to a challenging condition between groups. Results showed a decrease in N1 amplitude from zero- to two-back in NH controls. In CI users, the N1 responses were smaller and minimally affected by the working memory load. This different pattern of N1 response could be related to allocation of attentional resources since the N1 wave is modulated by attention [106–110]. For instance, CI users may have engaged their attentional resources similarly for both conditions given the impoverished auditory input. Conversely, the decrease in N1 amplitude in NH controls may reflect an adaption to the increase in cognitive demand [111–115]. As the two-back requires maintenance of attention on both the current and preceding stimuli, perhaps attentional resources towards the current stimuli are diminished to retain the previous numbers presented.

While CI users showed similar N1 amplitudes between conditions, older CI users showed an increase in N1 amplitude from zero- to two-back. Similar results of larger AEPs are observed in older NH adults compared to younger ones [116], reflecting an increased allocation of attention toward auditory stimuli and evidence for deficits in attentional control. Although we did not observe an age effect in NH controls, these results imply that older CI users are utilizing more attention to adapt to increased task demands. Younger CI users exhibited patterns similar to NH controls; however, the N1 amplitude difference between conditions is still lower in younger CI users. Therefore, over time, they may develop a pattern of activity similar to the older CI users.

### Theta ERS differences in CI users

We compared theta and alpha/beta power after the two-digit number presentation to investigate cognitive differences between CI users and NH controls. In CI users, the theta ERS power during the two-back was reduced compared to NH controls, possibly reflecting difficulty mobilizing resources to encode and maintain the constant stream of auditory stimuli. Contrary to previous studies, no difference in frontal theta was observed between conditions in CI users and NH controls [117–120]. However, a difference in theta between groups was only observed in the two-back condition, we can assume that CI users could not adapt to the more difficult task compared to NH controls and perhaps reached supra-capacity levels, resulting in a diminished response. Evidence suggests that the frontal theta ERS activity may index working memory processes. Higher power is observed when encoding multiple sequential items in memory [121] and when using executive functioning to control attentional resources [43,122,123].

Prior research shows that older adults with mild cognitive impairment, assessed with the Mini-Mental State Examination [124], exhibited lower theta power while performing n-back tasks than those without clinically significant cognitive problems [45,48]. Similarly, CI users may be displaying signs of impairment in auditory memory encoding. This is further substantiated by the fact that older CI users showed lower theta power than younger CI users. This relationship between aging and theta ERS is commonly observed in working memory studies [49,100,125,126], suggesting an age-related deficiency in allocating the necessary resources towards encoding auditory stimuli into memory. However, the current study did not observe this relationship with NH controls. Possibilities for the diminished theta ERS are: 1) use of different cognitive strategies, 2) increased task difficulty [127] and 3) increased fatigue while time on task increases [35].

The first and third explanations are more likely as no differences in theta were observed between conditions. The first possibility suggests that CI users might rely on other methods of encoding verbal stimuli such as greater reliance on visual cues for speech processing as a compensatory strategy for their difficulties in auditory processing. Further research is required to determine if the reduced theta is a result of the physical settings of the CI or a consequence of neural plasticity when auditory processing is difficult. However, one study investigating the encoding of visual stimuli in adult CI users observed no differences in theta activity during the encoding phase suggesting that their visual encoding processes are intact [34]. The third possibility suggests that the diminished theta might reflect a decline of cognitive resources (specifically in older CI users) perhaps due to allocating more attentional resources than NH controls as the task progresses which would corroborate the reports of fatigue commonly observed in the CI population.

Additionally, the greater theta ERS activity in the right frontal cortex (comprised of the middle and inferior frontal gyri) was related to lower SIN scores and slower RTs in CI users; however, in NH controls, a greater theta ERS in this area trended towards faster RTs. The right middle and IFG are involved in Go/No-Go tasks and are commonly associated with reorienting attention and response inhibition, respectively [128]. In light of these findings, the NH controls could have been faster at identifying the target because they are reorienting their attention to the following two-digit number after responding as indexed by the right frontal cortex activity. Alternatively, CI users with lower SIN scores and slower RTs were more likely to withhold responding to a target, possibly due to target uncertainty associated with deficits in encoding the two-digit stimuli. Greater theta ERS activity in the left IFG was correlated with lower performance for the CI users and decreased auditory N1 responses for NH controls. Since the left IFG is involved in speech perception when stimuli are degraded [57,58], CI users with lower performance and NH controls with lower auditory responsiveness to the stimuli perhaps engaged a compensatory strategy through the left IFG activation to encode the stimuli.

### Alpha/Beta activity differences in CI users

The role of alpha/beta activity in working memory is commonly studied where desynchrony during encoding and its modulation by memory load suggests an increased use of attentional resources [38,50–52,54,117]. However, alpha/beta synchrony is thought to play a role in maintaining stimuli and is suggested to represent cortical inhibition while protecting task-relevant information [129–132]. In the current study, we observed two different activity patterns in CI users and NH controls; all CI participants displayed an alpha/beta ERD, while NH controls showed both ERS and ERD. This may indicate that CI users engage more attentional resources to encode auditory stimuli while NH controls use more resources to maintain the stimuli in memory.

This possibility is supported by the observed reduced frontotemporal connectivity in CI users compared to NH controls. Comprised of the left STG and the left IFG, these areas are involved in the phonological loop for verbal rehearsal [133,134] therefore, the NH controls might subvocally maintain the stimuli in memory during the alpha/beta time window. In a previous study, after the visual presentation of verbal stimuli (numbers and letters), CI users displayed an increased left IFG and left STG connectivity compared to NH controls [34], indicating that when visual stimuli are presented, CI users can utilize this connectivity for maintaining items in memory. However, when auditory stimuli are presented, CI users might prioritize the left IFG for speech perception resulting in delays in encoding.

To corroborate this, CI users with more delayed RTs displayed greater alpha/beta ERD in the left IFG, and those with faster RTs displayed an ERS. Alpha/beta ERS or ERD largely depends on the listener’s cognitive strategies toward performing the task [126]. Listening strategies also change as listening effort increases [33] and as the signal-to-noise ratio of auditory stimuli decreases [135]; both of which are depicted as inverted U-shaped functions. Alpha/beta ERD or low ERS is observed when participants report lower listening effort or during an easier listening condition. As listening effort and difficulty increase, alpha power increases, reaching a limit at the intermediate level and then decreasing back to an ERD or a lower ERS. The latter decrease in ERS in response to increased listening effort and greater auditory degradation reflects an attentional switch towards actively processing the target stimuli. This may imply that CI users use more listening effort to disambiguate auditory stimuli, thereby delaying the identification of targets in the current study.

### Implications, limitations and future directions

This study demonstrates several neural differences between CI users compared to NH controls while performing an auditory working memory task providing insight into the cognitive effects of listening through a CI. The results imply that CI users utilize greater attentional resources than NH controls to potentially compensate for their impoverished auditory input (AEP amplitudes) and deficiency in auditory memory encoding (reduced theta ERS). The increased attentional effort dedicated to stimulus encoding may leave inadequate resources for maintenance, resulting in the delayed cognitive processing of target stimuli. This study also provides insights into aging with a CI as older CI users allocate more attentional resources during encoding than younger users, potentially contributing to delays in identifying the targets. Consequences of chronic increased cognitive demand while listening to speech and the subsequent reduction in resource availability might lead to psychological effects such as social isolation, depression, and anxiety [7–17]. Over time, these individuals might experience cognitive decline and dementia [1,136–138]. However, future studies will have to confirm that.

A limitation of this study is its ecological validity. Participants encoded numbers rather than words and sentences and the study was administered in a soundproof room with no background noise. While the n-back resembles the template of encoding speech in the real world (encoding, maintaining, and retrieving continuous stimuli), future studies should use a more realistic environment and stimuli. Another limitation is interpretation of the aging effect using response times, ΔN1, and theta activity. As no significant relationships were observed with the neural results and age of implantation, duration of deafness and duration of implantation, we concluded that the significant correlations with age were a result of aging with a CI. However, other factors might influence the observed results such as the CI group sample being small and heterogeneous, Menière’s disease in only two older CI users which may contribute to cognitive difficulties [138] and no use of cognitive screening tests to measure for cognitive impairment therefore, it is unknown if cognitive impairment was present in the older CI users.

## Conclusions

We investigated neural activity in CI users and NH controls while performing an auditory working memory task. Resulting differences between groups indicate a deficiency in auditory memory encoding in CI users, potentially resulting in greater recruitment of attentional resources for stimulus encoding, leaving an inadequate amount of resources for maintaining stimuli in working memory. The results of greater attentional resource recruitment were more evident in older CI users and related to more delayed identification of target stimuli. These findings clarify the relationship between auditory processing and cognitive ability in CI users and provide potential effects on aging with a CI.

## Supporting information

S1 Fig Time-frequency representations of correct and incorrect trials. Correct trial time-frequency representations are displayed for CI users during the A) zero- and B) two-back and for the NH controls during the C) zero- and D) two-back. Incorrect trial time-frequency representations are displayed for CI users during the E) zero- and F) two-back and for the NH controls during the G) zero- and H) two-back.
(ZIP)

S1 Data CI_NH_data. (XLSX)

S1 File All trials. (ZIP)

S2 File Correct_incorrect_trials. (ZIP)

## References

[1] Livingston G, Sommerlad A, Orgeta V. The lancet international commission on dementia prevention and care. Lancet. 2017;390:2673–734. 28735855 10.1016/S0140-6736(17)31363-6

[2] Campbell J, Sharma A. Compensatory changes in cortical resource allocation in adults with hearing loss. Front Syst Neurosci. 2013;7:71. doi: 10.3389/fnsys.2013.00071 24478637 PMC3905471

[3] Peelle JE, Troiani V, Grossman M, Wingfield A. Hearing loss in older adults affects neural systems supporting speech comprehension. J Neurosci. 2011;31(35):12638–43. doi: 10.1523/JNEUROSCI.2559-11.2011 21880924 PMC3175595

[4] Stahl SM. Does treating hearing loss prevent or slow the progress of dementia? Hearing is not all in the ears, but who’s listening?. CNS Spectr. 2017;22(3):247–50. doi: 10.1017/S1092852917000268 28376938

[5] Wayne RV, Johnsrude IS. A review of causal mechanisms underlying the link between age-related hearing loss and cognitive decline. Ageing Res Rev. 2015;23(Pt B):154–66. doi: 10.1016/j.arr.2015.06.002 26123097

[6] Rönnberg J, Lunner T, Zekveld A. The Ease of Language Understanding (ELU) model: theoretical, empirical, and clinical advances. Front Syst Neurosci. 2013;7:1–17. 23874273 10.3389/fnsys.2013.00031 PMC3710434

[7] Akeroyd MA. Are individual differences in speech reception related to individual differences in cognitive ability? A survey of twenty experimental studies with normal and hearing-impaired adults. Int J Audiol. 2008;47 Suppl 2:S53-71. doi: 10.1080/14992020802301142 19012113

[8] Fritze T, Teipel S, Óvári A, Kilimann I, Witt G, Doblhammer G. Hearing impairment affects dementia incidence. an analysis based on longitudinal health claims data in Germany. PLoS One. 2016;11(7):e0156876. doi: 10.1371/journal.pone.0156876 27391486 PMC4938406

[9] Fulton SE, Lister JJ. Mechanisms of the Hearing – Cognition Relationship. Semin Hear. 2015;36:140–9. 27516714 10.1055/s-0035-1555117 PMC4906307

[10] Gallacher J, Ilubaera V, Ben-Shlomo Y, et al. Auditory threshold, phonologic demand, and incident dementia. Neurology. 2012;79:1583–90. 23019269 10.1212/WNL.0b013e31826e263d

[11] Golub JS. Brain changes associated with age-related hearing loss. Curr Opin Otolaryngol Head Neck Surg. 2017;25(5):347–52. doi: 10.1097/MOO.0000000000000387 28661962

[12] Gurgel RK, Ward PD, Schwartz S, Norton MC, Foster NL, Tschanz JT. Relationship of hearing loss and dementia: a prospective, population-based study. Otol Neurotol. 2014;35(5):775–81. doi: 10.1097/MAO.0000000000000313 24662628 PMC4024067

[13] Lin FR, Yaffe K, Xia J, Xue Q-L, Harris TB, Purchase-Helzner E, et al. Hearing loss and cognitive decline in older adults. JAMA Intern Med. 2013;173(4):293–9. doi: 10.1001/jamainternmed.2013.1868 23337978 PMC3869227

[14] Lin FR, Ferrucci L, An Y, Goh JO, Doshi J, Metter EJ, et al. Association of hearing impairment with brain volume changes in older adults. Neuroimage. 2014;90:84–92. doi: 10.1016/j.neuroimage.2013.12.059 24412398 PMC3951583

[15] Loughrey DG, Kelly ME, Kelley GA, Brennan S, Lawlor BA. Association of age-related hearing loss with cognitive function, cognitive impairment, and dementia: a systematic review and meta-analysis. JAMA Otolaryngol Head Neck Surg. 2018;144(2):115–26. doi: 10.1001/jamaoto.2017.2513 29222544 PMC5824986

[16] Strawbridge WJ, Wallhagen MI, Shema SJ. Negative consequences of hearing impairment in old age: A longitudinal analysis. Gerontologist. 2000;40:320–6. 10853526 10.1093/geront/40.3.320

[17] Wingfield A. Evolution of models of working memory and cognitive resources. Ear Hear. 2016;37 Suppl 1:35S-43S. doi: 10.1097/AUD.0000000000000310 27355768

[18] Amieva H, Ouvrard C, Giulioli C, Meillon C, Rullier L, Dartigues J-F. Self-reported hearing loss, hearing aids, and cognitive decline in elderly adults: a 25-year study. J Am Geriatr Soc. 2015;63(10):2099–104. doi: 10.1111/jgs.13649 26480972

[19] Deal JA, Sharrett AR, Albert MS, Coresh J, Mosley TH, Knopman D, et al. Hearing impairment and cognitive decline: a pilot study conducted within the atherosclerosis risk in communities neurocognitive study. Am J Epidemiol. 2015;181(9):680–90. doi: 10.1093/aje/kwu333 25841870 PMC4408947

[20] Sugiura S, Nishita Y, Uchida Y, Shimono M, Suzuki H, Teranishi M, et al. Longitudinal associations between hearing aid usage and cognition in community-dwelling Japanese older adults with moderate hearing loss. PLoS One. 2021;16(10):e0258520. doi: 10.1371/journal.pone.0258520 34644353 PMC8513843

[21] Maharani A, Dawes P, Nazroo J, Tampubolon G, Pendleton N, SENSE-Cog WP1 group. Longitudinal relationship between hearing aid use and cognitive function in older Americans. J Am Geriatr Soc. 2018;66(6):1130–6. doi: 10.1111/jgs.15363 29637544

[22] Mosnier I, Bebear J-P, Marx M, Fraysse B, Truy E, Lina-Granade G, et al. Improvement of cognitive function after cochlear implantation in elderly patients. JAMA Otolaryngol Head Neck Surg. 2015;141(5):442–50. doi: 10.1001/jamaoto.2015.129 25763680

[23] Cosetti MK, Pinkston JB, Flores JM, Friedmann DR, Jones CB, Roland JT Jr, et al. Neurocognitive testing and cochlear implantation: insights into performance in older adults. Clin Interv Aging. 2016;11:603–13. doi: 10.2147/CIA.S100255 27274210 PMC4869653

[24] Claes AJ, Van de Heyning P, Gilles A. Impaired cognitive functioning in cochlear implant recipients over the age of 55 years: A cross-sectional study using the Repeatable Battery for the Assessment of Neuropsychological Status for Hearing-impaired individuals (RBANS-H). Front Neurosci. 2018;12:1–8. 30197584 10.3389/fnins.2018.00580 PMC6117382

[25] Andries E, Bosmans J, Engelborghs S, Cras P, Vanderveken OM, Lammers MJW, et al. Evaluation of cognitive functioning before and after cochlear implantation in adults aged 55 years and older at risk for mild cognitive impairment. JAMA Otolaryngol Head Neck Surg. 2023;149(4):310–6. doi: 10.1001/jamaoto.2022.5046 36795400 PMC9936380

[26] Völter C, Götze L, Kamin ST, Haubitz I, Dazert S, Thomas JP. Can cochlear implantation prevent cognitive decline in the long-term follow-up?. Front Neurol. 2022;13:1009087. doi: 10.3389/fneur.2022.1009087 36341108 PMC9631779

[27] Mosnier I, Vanier A, Bonnard D, Lina-Granade G, Truy E, Bordure P, et al. Long-term cognitive prognosis of profoundly deaf older adults after hearing rehabilitation using cochlear implants. J Am Geriatr Soc. 2018;66(8):1553–61. doi: 10.1111/jgs.15445 30091185

[28] Claes AJ, Van de Heyning P, Gilles A, Hofkens-Van den Brandt A, Van Rompaey V, Mertens G. Impaired cognitive functioning in cochlear implant recipients over the age of 55 years: a cross-sectional study using the Repeatable Battery for the Assessment of Neuropsychological Status for Hearing-Impaired Individuals (RBANS-H). Front Neurosci. 2018;12:580. doi: 10.3389/fnins.2018.00580 30197584 PMC6117382

[29] Huber M, Roesch S, Pletzer B, Lukaschyk J, Lesinski-Schiedat A, Illg A. Cognition in older adults with severe to profound sensorineural hearing loss compared to peers with normal hearing for age. Int J Audiol. 2020;59(4):254–62. doi: 10.1080/14992027.2019.1687947 31718333

[30] Angelo TCS de, Moret ALM, Costa OA da, Nascimento LT, Alvarenga K de F. Quality of life in adult cochlear implant users. Codas. 2016;28(2):106–12. doi: 10.1590/2317-1782/20162015097 27191872

[31] Dimitrijevic A, Smith ML, Kadis DS, Moore DR. Neural indices of listening effort in noisy environments. Sci Rep. 2019;9(1):11278. doi: 10.1038/s41598-019-47643-1 31375712 PMC6677804

[32] Kessler M, Schierholz I, Mamach M, Wilke F, Hahne A, Büchner A, et al. Combined brain-perfusion SPECT and EEG measurements suggest distinct strategies for speech comprehension in CI users with higher and lower performance. Front Neurosci. 2020;14:787. doi: 10.3389/fnins.2020.00787 32848560 PMC7431776

[33] Paul BT, Chen J, Le T, Lin V, Dimitrijevic A. Cortical alpha oscillations in cochlear implant users reflect subjective listening effort during speech-in-noise perception. PLoS One. 2021;16(7):e0254162. doi: 10.1371/journal.pone.0254162 34242290 PMC8270138

[34] Prince P, Paul BT, Chen J. Neural correlates of visual stimulus encoding and verbal working memory differ between cochlear implant users and normal-hearing controls. European Journal of Neuroscience. 2021;54:5016–37. 34146363 10.1111/ejn.15365 PMC8457219

[35] Wascher E, Rasch B, Sänger J, Hoffmann S, Schneider D, Rinkenauer G, et al. Frontal theta activity reflects distinct aspects of mental fatigue. Biol Psychol. 2014;96:57–65. doi: 10.1016/j.biopsycho.2013.11.010 24309160

[36] Kawasaki M, Yamaguchi Y. Effects of subjective preference of colors on attention-related occipital theta oscillations. Neuroimage. 2012;59(1):808–14. doi: 10.1016/j.neuroimage.2011.07.042 21820064

[37] Kawasaki M, Yamaguchi Y. Frontal theta and beta synchronizations for monetary reward increase visual working memory capacity. Soc Cogn Affect Neurosci. 2013;8(5):523–30. doi: 10.1093/scan/nss027 22349800 PMC3682435

[38] Dong S, Reder LM, Yao Y, Liu Y, Chen F. Individual differences in working memory capacity are reflected in different ERP and EEG patterns to task difficulty. Brain Res. 2015;1616:146–56. doi: 10.1016/j.brainres.2015.05.003 25976774

[39] Moran RJ, Campo P, Maestu F. Peak frequency in the theta and alpha bands correlates with human working memory capacity. 2010;4:1–12. 10.3389/fnhum.2010.00200 PMC3009479 21206531

[40] Zakrzewska MZ, Brzezicka A. Working memory capacity as a moderator of load-related frontal midline theta variability in Sternberg task. Psychological Bulletin. 2014;8:1–7. 10.3389/fnhum.2014.00399 PMC4047791 24936180

[41] Kwon G, Lim S, Kim M-Y, Kwon H, Lee Y-H, Kim K, et al. Individual differences in oscillatory brain activity in response to varying attentional demands during a word recall and oculomotor dual task. Front Hum Neurosci. 2015;9:381. doi: 10.3389/fnhum.2015.00381 26175681 PMC4484223

[42] Pavlov YG, Kotchoubey B. EEG correlates of working memory performance in females. BMC Neurosci. 2017;18(1):26. doi: 10.1186/s12868-017-0344-5 28193169 PMC5307759

[43] Maurer U, Brem S, Liechti M, Maurizio S, Michels L, Brandeis D. Frontal midline theta reflects individual task performance in a working memory task. Brain Topogr. 2015;28(1):127–34. doi: 10.1007/s10548-014-0361-y 24687327

[44] Hsieh L-T, Ekstrom AD, Ranganath C. Neural oscillations associated with item and temporal order maintenance in working memory. J Neurosci. 2011;31(30):10803–10. doi: 10.1523/JNEUROSCI.0828-11.2011 21795532 PMC3164584

[45] Deiber M-P, Ibañez V, Missonnier P, Herrmann F, Fazio-Costa L, Gold G, et al. Abnormal-induced theta activity supports early directed-attention network deficits in progressive MCI. Neurobiol Aging. 2009;30(9):1444–52. doi: 10.1016/j.neurobiolaging.2007.11.021 18179844

[46] Deiber M-P, Meziane HB, Hasler R, Rodriguez C, Toma S, Ackermann M, et al. Attention and working memory-related EEG markers of subtle cognitive deterioration in healthy elderly individuals. J Alzheimers Dis. 2015;47(2):335–49. doi: 10.3233/JAD-150111 26401557

[47] Fraga FJ, Mamani GQ, Johns E, Tavares G, Falk TH, Phillips NA. Early diagnosis of mild cognitive impairment and Alzheimer’s with event-related potentials and event-related desynchronization in N-back working memory tasks. Comput Methods Programs Biomed. 2018;164:1–13. doi: 10.1016/j.cmpb.2018.06.011 30195417

[48] Missonnier P, Gold G, Herrmann FR. Decreased theta event-related synchronization during working memory activation is associated with progressive mild cognitive impairment. Dement Geriatr Cogn Disord. 2006;22:250–9. 16902280 10.1159/000094974

[49] Nowak K, Costa-Faidella J, Dacewicz A, Escera C, Szelag E. Altered event-related potentials and theta oscillations index auditory working memory deficits in healthy aging. Neurobiol Aging. 2021;108:1–15. doi: 10.1016/j.neurobiolaging.2021.07.019 34464912

[50] Hjortkjaer J, Märcher-Rørsted J, Fuglsang SA, Dau T. Cortical oscillations and entrainment in speech processing during working memory load. Eur J Neurosci. 2020;51(5):1279–89. doi: 10.1111/ejn.13855 29392835 PMC7155003

[51] Costers L, Van Schependom J, Laton J, Baijot J, Sjøgård M, Wens V, et al. Spatiotemporal and spectral dynamics of multi-item working memory as revealed by the n-back task using MEG. Hum Brain Mapp. 2020;41(9):2431–46. doi: 10.1002/hbm.24955 32180307 PMC7267970

[52] Wang S, Gwizdka J, Chaovalitwongse WA. Using wireless EEG signals to assess memory workload in the n-back task. IEEE Transactions on Human-Machine Systems. 2016;46:424–35.

[53] Lei S, Roetting M. Influence of task combination on EEG spectrum modulation for driver workload estimation. Hum Factors. 2011;53:168–79. 21702334 10.1177/0018720811400601

[54] Scharinger C, Soutschek A, Schubert T. Comparison of the working memory load in N-back and working memory span tasks by means of EEG frequency band power and P300 amplitude. Front Hum Neurosci. 2017;11:1–19. 28179880 10.3389/fnhum.2017.00006 PMC5263141

[55] Jaeggi SM, Buschkuehl M, Jonides J, Perrig WJ. Improving fluid intelligence with training on working memory. Proc Natl Acad Sci U S A. 2008;105(19):6829–33. doi: 10.1073/pnas.0801268105 18443283 PMC2383929

[56] Dimitrijevic A, Smith ML, Kadis DS, Moore DR. Cortical alpha oscillations predict speech intelligibility. Front Hum Neurosci. 2017;11:88. doi: 10.3389/fnhum.2017.00088 28286478 PMC5323373

[57] Alain C, Du Y, Bernstein LJ, Barten T, Banai K. Listening under difficult conditions: An activation likelihood estimation meta-analysis. Hum Brain Mapp. 2018;39(7):2695–709. doi: 10.1002/hbm.24031 29536592 PMC6866419

[58] Rovetti J, Goy H, Zara M, et al. Reduced Semantic Context and Signal-to-Noise Ratio Increase Listening Effort As Measured Using Functional Near-Infrared Spectroscopy. 2022; 836–48. 10.1097/AUD.0000000000001137 34623112

[59] Owen AM, McMillan KM, Laird AR, Bullmore E. N-back working memory paradigm: a meta-analysis of normative functional neuroimaging studies. Hum Brain Mapp. 2005;25(1):46–59. doi: 10.1002/hbm.20131 15846822 PMC6871745

[60] Spahr AJ, Dorman MF. Effects of minimum stimulation settings for the Med El Tempo+ speech processor on speech understanding. Ear Hear. 2005;26(4 Suppl):2S-6S. doi: 10.1097/00003446-200508001-00002 16082262

[61] Brain Products GmbH. BrainVision Analyzer.

[62] Gilley PM, Sharma A, Dorman M, et al. Minimization of cochlear implant stimulus artifact in cortical auditory evoked potentials. Clinical Neurophysiology 2006; 117: 1772–82. 16807102 10.1016/j.clinph.2006.04.018

[63] Castañeda-Villa N, James CJ. Independent component analysis for auditory evoked potentials and cochlear implant artifact estimation. IEEE Trans Biomed Eng. 2011;58(2):348–54. doi: 10.1109/TBME.2010.2072957 20813628

[64] Delorme A, Makeig S. EEGLAB: an open source toolbox for analysis of single-trial EEG dynamics including independent component analysis. J Neurosci Methods. 2004;134(1):9–21. doi: 10.1016/j.jneumeth.2003.10.009 15102499

[65] Oostenveld R, Fries P, Maris E, Schoffelen J-M. FieldTrip: Open source software for advanced analysis of MEG, EEG, and invasive electrophysiological data. Comput Intell Neurosci. 2011;2011:156869. doi: 10.1155/2011/156869 21253357 PMC3021840

[66] Fieldtrip. Does it make sense to subtract the ERP prior to time frequency analysis, to distinguish evoked from induced power?. 2020. Available fom: https://www.fieldtriptoolbox.org/faq/evoked_vs_induced/

[67] David O, Kilner JM, Friston KJ. Mechanisms of evoked and induced responses in MEG/EEG. Neuroimage. 2006;31(4):1580–91. doi: 10.1016/j.neuroimage.2006.02.034 16632378

[68] Percival D, Walden A. Spectral Analysis for Physical Applications. Cambridge: Cambridge University Press; 1993.

[69] Mitra PP, Pesaran B. Analysis of dynamic brain imaging data. Biophys J. 1999;76(2):691–708. doi: 10.1016/S0006-3495(99)77236-X 9929474 PMC1300074

[70] Gramfort A, Papadopoulo T, Olivi E, Clerc M. OpenMEEG: opensource software for quasistatic bioelectromagnetics. Biomed Eng Online. 2010;9:45. doi: 10.1186/1475-925X-9-45 20819204 PMC2949879

[71] Kybic J, Clerc M, Abboud T. A common formalism for the integral formulations of the forward EEG problem. IEEE Trans Med Imaging. 2005;24:12–28. 15638183 10.1109/tmi.2004.837363

[72] Palmero-Soler E, Dolan K, Hadamschek V, Tass PA. swLORETA: a novel approach to robust source localization and synchronization tomography. Phys Med Biol. 2007;52(7):1783–800. doi: 10.1088/0031-9155/52/7/002 17374911

[73] Tadel F, Baillet S, Mosher JC, Pantazis D, Leahy RM. Brainstorm: a user-friendly application for MEG/EEG analysis. Comput Intell Neurosci. 2011;2011:879716. doi: 10.1155/2011/879716 21584256 PMC3090754

[74] Desikan RS, Ségonne F, Fischl B, Quinn BT, Dickerson BC, Blacker D, et al. An automated labeling system for subdividing the human cerebral cortex on MRI scans into gyral based regions of interest. Neuroimage. 2006;31(3):968–80. doi: 10.1016/j.neuroimage.2006.01.021 16530430

[75] Vinck M, Oostenveld R, van Wingerden M, Battaglia F, Pennartz CMA. An improved index of phase-synchronization for electrophysiological data in the presence of volume-conduction, noise and sample-size bias. Neuroimage. 2011;55(4):1548–65. doi: 10.1016/j.neuroimage.2011.01.055 21276857

[76] Palva JM, Wang SH, Palva S. Ghost interactions in MEG/EEG source space: A note of caution on inter-areal coupling measures. Neuroimage. 2018;173:632–43. 29477441 10.1016/j.neuroimage.2018.02.032

[77] Kumar S, Joseph S, Gander PE, Barascud N, Halpern AR, Griffiths TD. A brain system for auditory working memory. J Neurosci. 2016;36(16):4492–505. doi: 10.1523/JNEUROSCI.4341-14.2016 27098693 PMC4837683

[78] Xu K, Duann J-R. Brain connectivity in the left frontotemporal network dynamically modulated by processing difficulty: Evidence from Chinese relative clauses. PLoS One. 2020;15(4):e0230666. doi: 10.1371/journal.pone.0230666 32271773 PMC7144993

[79] Rodrigues de Almeida L, Pope PA, Hansen PC. Motor theory modulated by task load: Effects of tDCS over the LSTG on connectivity patterns for phonological processing. J Neurolinguistics. 2021;58:100984.

[80] Xu K, Wu DH, Duann J-R. Dynamic brain connectivity attuned to the complexity of relative clause sentences revealed by a single-trial analysis. Neuroimage. 2020;217:116920. doi: 10.1016/j.neuroimage.2020.116920 32422404

[81] Choi MH, Kim HS, Chung SC. Evaluation of effective connectivity between brain areas activated during simulated driving using dynamic causal modeling. Front Behav Neurosci. 2020;14:1–10. 33173471 10.3389/fnbeh.2020.00158 PMC7538660

[82] Teghipco A, Hussain A, Tivarus ME. Disrupted functional connectivity affects resting state based language lateralization. Neuroimage Clin. 2016;12:910–27. doi: 10.1016/j.nicl.2016.10.015 27882297 PMC5114586

[83] Team Rs. RStudio: Integrated Development for R. RStudio, 2020. http://www.rstudio.com/

[84] Benjamini Y, Hochberg Y. Controlling the false discovery rate: a practical and powerful approach to multiple testing. Journal of the Royal Statistical Society Series B (Methodological). 1995;57:289–300.

[85] Revelle W. Psych: Procedures for Psychological, Psychometric, and Personality Research.

[86] Rousselet GA, Pernet CR. Improving standards in brain-behavior correlation analyses. Front Hum Neurosci. 2012;6:119. doi: 10.3389/fnhum.2012.00119 22563313 PMC3342588

[87] Amon MJ, Bertenthal BI. Auditory versus visual stimulus effects on cognitive performance during the N-back task. In: Proceedings of the 40th Annual Meeting of the Cognitive Science Society, 2018. 1304–9.

[88] Rovetti J, Goy H, Pichora-Fuller MK, Russo FA. Functional near-infrared spectroscopy as a measure of listening effort in older adults who use hearing aids. Trends Hear. 2019;23:2331216519886722. doi: 10.1177/2331216519886722 31722613 PMC6856975

[89] Tao D, Deng R, Jiang Y, Galvin JJ 3rd, Fu Q-J, Chen B. Contribution of auditory working memory to speech understanding in mandarin-speaking cochlear implant users. PLoS One. 2014;9(6):e99096. doi: 10.1371/journal.pone.0099096 24921934 PMC4055598

[90] Hamdy M, El Shennawy A, Mostafa N, Hamdy HS. Working memory and listening fatigue in cochlear implantation. Hearing Balance and Communication. 2023;21(4):246–54. doi: 10.1080/21695717.2023.2188813

[91] Moberly AC, Harris MS, Boyce L, Nittrouer S. Speech recognition in adults with cochlear implants: the effects of working memory, phonological sensitivity, and aging. J Speech Lang Hear Res. 2017;60(4):1046–61. doi: 10.1044/2016_JSLHR-H-16-0119 28384805 PMC5548076

[92] Nagels L, Bastiaanse R, Başkent D, Wagner A. Individual differences in lexical access among cochlear implant users. J Speech Lang Hear Res. 2019;63(1):286–304. doi: 10.1044/2019_JSLHR-19-00192 31855606

[93] Amenta S, Artesini L, Musola D, Frau GN, Vespignani F, Pavani F. Probing language processing in cochlear implant users with visual word recognition: effects of lexical and orthographic word properties. Language, Cognition and Neuroscience. 2020;36(2):187–98. doi: 10.1080/23273798.2020.1804600

[94] Rönnberg J, Rudner M, Lunner T, Zekveld AA. When cognition kicks in: working memory and speech understanding in noise. Noise Health. 2010;12(49):263–9. doi: 10.4103/1463-1741.70505 20871181

[95] Mattys SL, Davis MH, Bradlow AR. Speech recognition in adverse conditions: a review. Lang Cogn Process. 2012;27:953–78.

[96] Ohlenforst B, Zekveld AA, Jansma EP, Wang Y, Naylor G, Lorens A, et al. Effects of hearing impairment and hearing aid amplification on listening effort: a systematic review. Ear Hear. 2017;38(3):267–81. doi: 10.1097/AUD.0000000000000396 28234670 PMC5405775

[97] Pals C, Sarampalis A, Beynon A, Stainsby T, Başkent D. Effect of spectral channels on speech recognition, comprehension, and listening effort in cochlear-implant users. Trends Hear. 2020;24:2331216520904617. doi: 10.1177/2331216520904617 32189585 PMC7082863

[98] Gajewski PD, Hanisch E, Falkenstein M, Thönes S, Wascher E. What does the n-back task measure as we get older? relations between working-memory measures and other cognitive functions across the lifespan. Front Psychol. 2018;9:2208. doi: 10.3389/fpsyg.2018.02208 30534095 PMC6275471

[99] Gajewski PD, Falkenstein M. Age-related effects on erp and oscillatory EEG-dynamics in a 2-back task. J Psychophysiol 2015; 28: 162–77.

[100] Bopp KL, Verhaeghen P. Aging and n-Back performance: a meta-analysis. J Gerontol B Psychol Sci Soc Sci. 2020;75(2):229–40. doi: 10.1093/geronb/gby024 31943115

[101] Moberly AC, Vasil KJ, Wucinich TL. How does aging affect recognition of spectrally degraded speech?. Laryngoscope. 2018;128. doi: 10.1002/lary.27457 PMC6572764 30325518

[102] Li MM, Moberly AC, Tamati TN. Factors affecting talker discrimination ability in adult cochlear implant users. J Commun Disord. 2022;99:106255. doi: 10.1016/j.jcomdis.2022.106255 35988314 PMC10659049

[103] Sladen DP, Zappler A. Older and younger adult cochlear implant users: speech recognition in quiet and noise, quality of life, and music perception. Am J Audiol. 2015;24(1):31–9. doi: 10.1044/2014_AJA-13-0066 25239296

[104] Burkhardt P, Müller V, Meister H, Weglage A, Lang-Roth R, Walger M, et al. Age effects on cognitive functions and speech-in-noise processing: An event-related potential study with cochlear-implant users and normal-hearing listeners. Front Neurosci. 2022;16:1005859. doi: 10.3389/fnins.2022.1005859 36620447 PMC9815545

[105] Mai A, Hillyard SA, Strauss DJ. Linear modeling of brain activity during selective attention to continuous speech: the critical role of the N1 effect in event-related potentials to acoustic edges. *bioRxiv*. Epub ahead of print 2023. doi: 10.1101/2023.07.14.548994 PMC12222594 40612893

[106] Fiedler L, Wöstmann M, Graversen C, Brandmeyer A, Lunner T, Obleser J. Single-channel in-ear-EEG detects the focus of auditory attention to concurrent tone streams and mixed speech. J Neural Eng. 2017;14(3):036020. doi: 10.1088/1741-2552/aa66dd 28384124

[107] Fiedler L, Wöstmann M, Herbst SK, Obleser J. Late cortical tracking of ignored speech facilitates neural selectivity in acoustically challenging conditions. Neuroimage. 2019;186:33–42. doi: 10.1016/j.neuroimage.2018.10.057 30367953

[108] Kaufman M, Zion Golumbic E. Listening to two speakers: Capacity and tradeoffs in neural speech tracking during Selective and Distributed Attention. Neuroimage. 2023;270:119984. doi: 10.1016/j.neuroimage.2023.119984 36854352

[109] Paredes-Gallardo A, Innes-Brown H, Madsen SMK, Dau T, Marozeau J. Auditory stream segregation and selective attention for cochlear implant listeners: evidence from behavioral measures and event-related potentials. Front Neurosci. 2018;12:581. doi: 10.3389/fnins.2018.00581 30186105 PMC6110823

[110] Ghani U, Signal N, Niazi IK, Taylor D. Efficacy of a single-task ERP measure to evaluate cognitive workload during a novel exergame. Front Hum Neurosci. 2021;15:742384. doi: 10.3389/fnhum.2021.742384 34566610 PMC8456040

[111] Ghani U, Signal N, Niazi IK, Taylor D. A novel approach to validate the efficacy of single task ERP paradigms to measure cognitive workload. Int J Psychophysiol. 2020;158:9–15. doi: 10.1016/j.ijpsycho.2020.09.007 33045292

[112] Muller-Gass A, Schröger E. Perceptual and cognitive task difficulty has differential effects on auditory distraction. Brain Res. 2007;1136(1):169–77. doi: 10.1016/j.brainres.2006.12.020 17223092

[113] Muller-Gass A, Macdonald M, Schröger E, Sculthorpe L, Campbell K. Evidence for the auditory P3a reflecting an automatic process: elicitation during highly-focused continuous visual attention. Brain Res. 2007;1170:71–8. doi: 10.1016/j.brainres.2007.07.023 17692834

[114] Deeny S, Chicoine C, Hargrove L, Parrish T, Jayaraman A. A simple ERP method for quantitative analysis of cognitive workload in myoelectric prosthesis control and human-machine interaction. PLoS One. 2014;9(11):e112091. doi: 10.1371/journal.pone.0112091 25402345 PMC4234315

[115] Alain C, Chow R, Lu J, Rabi R, Sharma VV, Shen D, et al. Aging enhances neural activity in auditory, visual, and somatosensory cortices: the common cause revisited. J Neurosci. 2022;42(2):264–75. doi: 10.1523/JNEUROSCI.0864-21.2021 34772740 PMC8802933

[116] Beckers L, Philips B, Huinck W, Mylanus E, Büchner A, Kral A. Auditory working memory in noise in cochlear implant users: insights from behavioural and neuronal measures. Hear Res. 2025;456:109167. doi: 10.1016/j.heares.2024.109167 39719815

[117] Ratcliffe O, Shapiro K, Staresina BP, et al. Article Fronto-medial theta coordinates posterior maintenance of working memory content ll ll Fronto-medial theta coordinates posterior maintenance of working memory content. Current Biology 2022;32:2121–29.e3. 35385693 10.1016/j.cub.2022.03.045 PMC9616802

[118] Eschmann KCJ, Bader R, Mecklinger A. Topographical differences of frontal-midline theta activity reflect functional differences in cognitive control abilities. Brain Cogn. 2018;123:57–64. doi: 10.1016/j.bandc.2018.02.002 29524859

[119] Scharinger C, Soutschek A, Schubert T, Gerjets P. Comparison of the working memory load in n-back and working memory span tasks by means of EEG frequency band power and P300 amplitude. Front Hum Neurosci. 2017;11:6. doi: 10.3389/fnhum.2017.00006 28179880 PMC5263141

[120] Hsieh L-T, Ranganath C. Frontal midline theta oscillations during working memory maintenance and episodic encoding and retrieval. Neuroimage. 2014;85 Pt 2(0 2):721–9. doi: 10.1016/j.neuroimage.2013.08.003 23933041 PMC3859771

[121] Fernández A, Pinal D, Díaz F. Working memory load modulates oscillatory activity and the distribution of fast frequencies across frontal theta phase during working memory maintenance. Neurobiol Learn Mem. 2021;183:107476. 34087476 10.1016/j.nlm.2021.107476

[122] Sauseng P, Griesmayr B, Freunberger R, Klimesch W. Control mechanisms in working memory: a possible function of EEG theta oscillations. Neurosci Biobehav Rev. 2010;34(7):1015–22. doi: 10.1016/j.neubiorev.2009.12.006 20006645

[123] Folstein MF, Folstein SE, McHugh PR. Mini-mental state: a practical method for grading the cognitive state of patients for the clinician. J Psychiatr Res. 1975;12:189–98. 1202204 10.1016/0022-3956(75)90026-6

[124] Kardos Z, Tóth B, Boha R, File B, Molnár M. Age-related changes of frontal-midline theta is predictive of efficient memory maintenance. Neuroscience. 2014;273:152–62. doi: 10.1016/j.neuroscience.2014.04.071 24846615

[125] Missonnier P, Herrmann FR, Rodriguez C, Deiber M-P, Millet P, Fazio-costa L, et al. Age-related differences on event-related potentials and brain rhythm oscillations during working memory activation. J Neural Transm (Vienna). 2011;118(6):945–55. doi: 10.1007/s00702-011-0600-2 21331458

[126] Ryan DB, Eckert MA, Sellers EW. Impact of effortful word recognition on supportive neural systems measured by alpha and theta power. Ear Hear. 2022;43:1549–62. 35363640 10.1097/AUD.0000000000001211

[127] Ball G, Stokes PR, Rhodes RA, Bose SK, Rezek I, Wink A-M, et al. Executive functions and prefrontal cortex: a matter of persistence?. Front Syst Neurosci. 2011;5:3. doi: 10.3389/fnsys.2011.00003 21286223 PMC3031025

[128] Klimesch W. EEG alpha and theta oscillations reflect cognitive and memory performance: a rKlimesch, W. (1999). EEG alpha and theta oscillations reflect cognitive and memory performance: a review and analysis. Brain Research Reviews, 29(2-3), 169–195. Brain Res Rev 1999;29:169–95. 10209231 10.1016/s0165-0173(98)00056-3

[129] Bonnefond M, Jensen O. Alpha oscillations serve to protect working memory maintenance against anticipated distracters. Curr Biol. 2012;22(20):1969–74. doi: 10.1016/j.cub.2012.08.029 23041197

[130] Bonnefond M, Jensen O. The role of gamma and alpha oscillations for blocking out distraction. Commun Integr Biol. 2013;6(1):e22702. doi: 10.4161/cib.22702 23802042 PMC3689574

[131] Wianda E, Ross B. The roles of alpha oscillation in working memory retention. Brain Behav. 2019;9(4):e01263. doi: 10.1002/brb3.1263 30887701 PMC6456781

[132] Fegen D, Buchsbaum BR, D’Esposito M. The effect of rehearsal rate and memory load on verbal working memory. Neuroimage. 2015;105:120–31. doi: 10.1016/j.neuroimage.2014.10.034 25467303 PMC4267698

[133] Herman AB, Houde JF, Vinogradov S, Nagarajan SS. Parsing the phonological loop: activation timing in the dorsal speech stream determines accuracy in speech reproduction. J Neurosci. 2013;33(13):5439–53. doi: 10.1523/JNEUROSCI.1472-12.2013 23536060 PMC3711632

[134] Wöstmann M, Lim S-J, Obleser J. The human neural alpha response to speech is a proxy of attentional control. Cereb Cortex. 2017;27(6):3307–17. doi: 10.1093/cercor/bhx074 28334352

[135] Lin FR, Ferrucci L, Metter EJ. Hearing loss and cognition in the baltimore longitudinal study of aging. Neuropsychology. 2011;25:763–70. 21728425 10.1037/a0024238 PMC3193888

[136] Lin FR, Metter EJ, O’Brien RJ, Resnick SM, Zonderman AB, Ferrucci L. Hearing loss and incident dementia. Arch Neurol. 2011;68(2):214–20. doi: 10.1001/archneurol.2010.362 21320988 PMC3277836

[137] Lopes L da C, Magaldi RM, Gândara MER, Reis AC de B, Jacob-Filho W. Prevalence of hearing impairment in patients with mild cognitive impairment. Dement Neuropsychol. 2007;1(3):253–9. doi: 10.1590/S1980-57642008DN10300006 29213398 PMC5619003

[138] Eraslan Boz H, Kırkım G, Koçoğlu K, Çakır Çetin A, Akkoyun M, Güneri EA, et al. Cognitive function in Meniere’s disease. Psychol Health Med. 2023;28(4):1076–86. doi: 10.1080/13548506.2022.2144637 36369758

