# Supplementary figures and images for: Effects of age on the neural correlates of auditory working memory in cochlear implant users
Source: PLoS One. 2025 Jun 25;20(6):e0325930. doi: 10.1371/journal.pone.0325930 (PMC12194263; doi:10.1371/journal.pone.0325930)

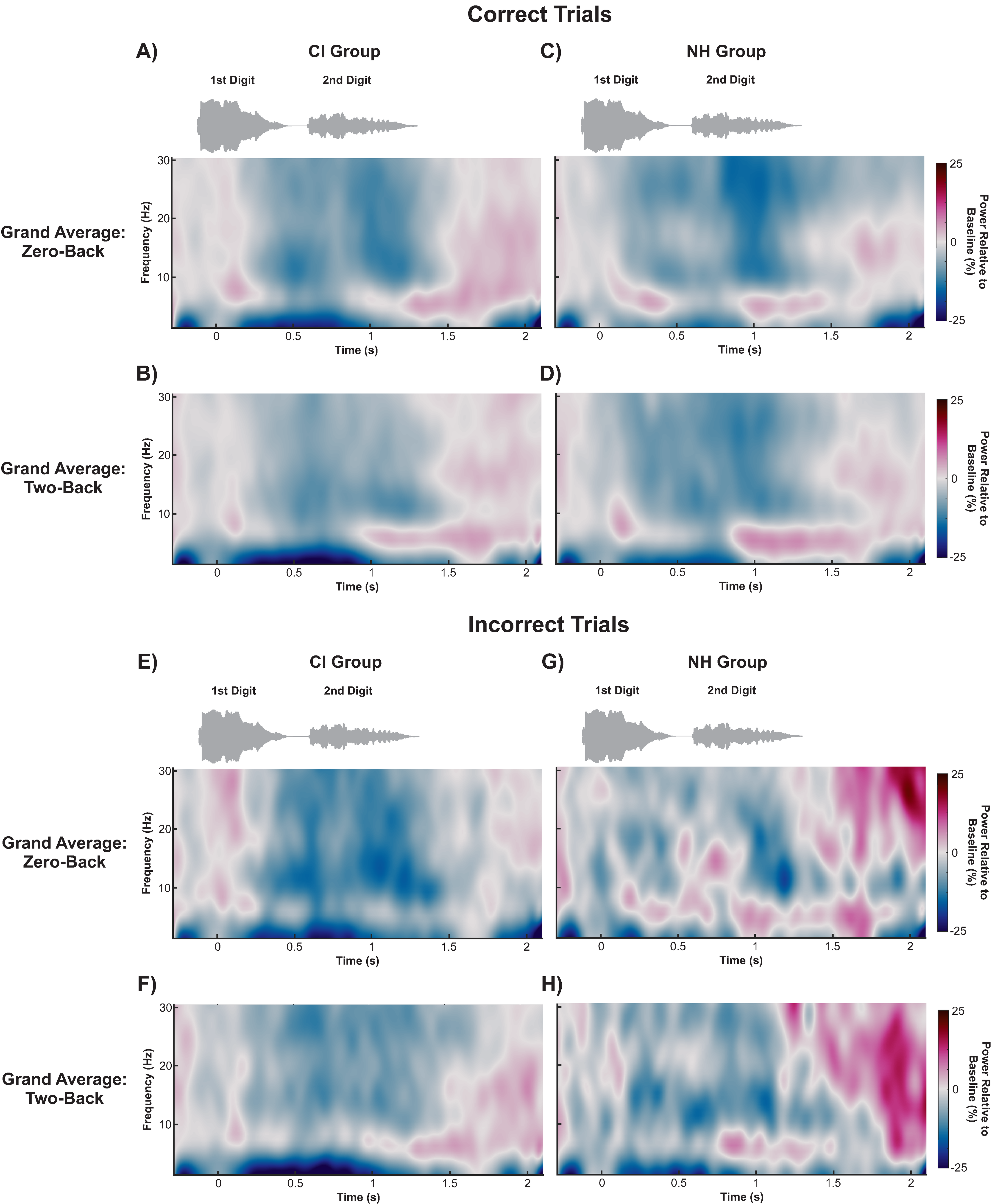

Supplement: S1 Fig — Correct trial time-frequency representations are displayed for CI users during the A) zero- and B) two-back and for the NH controls during the C) zero- and D) two-back. Incorrect trial time-frequency representations are displayed for CI users during the E) zero- and F) two-back and for the NH controls during the G) zero- and H) two-back. (ZIP) [file pone.0325930.s001.tif]
